# Dendrimer-modified gold nanorods as a platform for combinational gene therapy and photothermal therapy of tumors

**DOI:** 10.1186/s13046-021-02105-3

**Published:** 2021-09-27

**Authors:** Lili Ye, Yaoming Chen, Jizong Mao, Xiaotian Lei, Qian Yang, Chunhui Cui

**Affiliations:** 1 Department of Neuro-oncological Surgery, Zhujiang Hospital, Southern Medical University, Guangzhou, Guangdong Province China; 2 Department of General Surgery, Zhujiang Hospital, Southern Medical University, Guangzhou, Guangdong Province China

**Keywords:** Dendrimers, Gold nanorods, Gene delivery, Photothermal therapy, Cancer treatment

## Abstract

**Background:**

The exploitation of novel nanomaterials combining diagnostic and therapeutic functionalities within one single nanoplatform is challenging for tumor theranostics.

**Methods:**

We synthesized dendrimer-modified gold nanorods for combinational gene therapy and photothermal therapy (PTT) of colon cancer. Poly(amidoamine) dendrimers (PAMAM, G3) grafted gold nanorods were modified with GX1 peptide (a cyclic 7-mer peptide, CGNSNPKSC). The obtained Au NR@PAMAM-GX1 are proposed as a gene delivery vector to gene (FAM172A, regulates the proliferation and apoptosis of colon cancer cells) for the combination of photothermal therapy (PTT) and gene therapy of Colon cancer cells (HCT-8 cells). In addition, the CT imaging function of Au NR can provide imaging evidence for the diagnosis of colon cancer.

**Results:**

The results display that Au NR@PAMAM-GX1 can specifically deliver FAM172A to cancer cells with excellent transfection efficiency. The HCT-8 cells treated with the Au NR@PAMAM-GX1/FAM172A under laser irradiation have a viability of 20.45%, which is much lower than the survival rate of other single-mode PTT treatment or single-mode gene therapy. Furthermore, animal experiment results confirm that Au NR@PAMAM-GX1/FAM172A complexes can achieve tumor thermal imaging, targeted CT imaging, PTT and gene therapy after tail vein injection.

**Conclusion:**

Our findings demonstrate that the synthesized Au NR@PAMAM-GX1 offer a facile platform to exert antitumor and improve the diagnostic level of tumor.

**Supplementary Information:**

The online version contains supplementary material available at 10.1186/s13046-021-02105-3.

## Background

Traditional cancer treatments, such as surgery, chemotherapy and radiotherapy, have severe side effects. For example, forced resection may be life-threatening when tumor cells infiltrate surrounding tissues and organs or adhere to surrounding blood vessels and vital organs. Long-term chemotherapy can lead to tumor resistance to multiple drugs, making cancer treatment difficult. Moveover, killing cancer cells in the same way can also cause damage to healthy tissues or organs, causing side effects such as insomnia, vomiting, loss of appetite and leukopenia [1, 2]. Therefore, it is of great significance to develop a multi-functional nano-platform that integrates gene therapy and photothermal therapy (PTT).

Recently, PTT is an effective method for the treatment of tumors due to its low cost, good local treatment effect and little side effects [3–5]. PTT can induce tumor cell apoptosis or necrosis, thus inhibiting tumor growth by generating local heat called hyperthermia. For photothermal therapy of tumors, different nanoparticles have been designed and synthesized including carbon nanotubes [6], graphene oxide [7], and gold nanoparticles with different shapes (nanorods [8], nanostars [9], Au nano matryoshkas, nanochains [10]), Fe_3_O_4_ nanoparticles [11]. Although these nanoplatforms have improved the therapeutic performance, the synthesis processes is complex and time-consuming. Therefore, the development of simple and multifunctional nano-platforms as nanocarriers, photothermal agents and imaging agents is of great significance for synergistic phototherapy. Gold nanorods (Au NRs) are currently one of the most popular nanomaterials because of their excellent physical and chemical properties such as simple preparation, surface functionalization, low toxicity, good biocompatibility and rich biological activity [12–14]. Au NRs have been widely used as a nano-delivery nanoplatform for multimodal tumor therapy, such as drug therapy [15], photodynamic therapy [16] and gene delivery [17]. In particular, GNRs have different surface plasmon resonance (SPR) enhanced absorption bands in the near-infrared spectral region by adjusting the size and aspect ratio (aspect ratio) of GNRs [18]. Therefore, gold nanorods are considered as one of the most promising candidates for photothermal therapy (PTT) because blood and soft tissues have relatively low attenuation of the light.

However, the use of PTT alone has a limited light-penetration depth, which will substantially hinder the synergistic therapeutic efficiency of tumors in deeply located organs [19]. Gene therapy (GT) is a promising treatment with potential, which can introduce specific genes into target cells, restore defective genes or promote specific cell functions, thereby achieving long-term treatment [20, 21]. FAM172A is a functionally gene, which promotes proliferation and inhibites apoptosis in colon cancer cells. Prior study reported that STAT1, as a transcription factor, could bind to the minimum promoter region of FAM172A and upregulated the expression of FAM172A [22]. However, during the course of therapy, FAM172A suffers from a significant limitation (poor specificity, poor stability, short half- life and poor permeability), which is easily degraded in vivo via enzymes in the blood, skin, and other organs [23]. Additionally, circular heptapeptide GX1 (a cyclic 9-mer peptide, CGNSNPKSC), as a novel antitumor drug modification peptide, binds with great specificity to mouse tumor vascular endothelium, which had good potential for in vivo targeting [24].

**Scheme 1:**
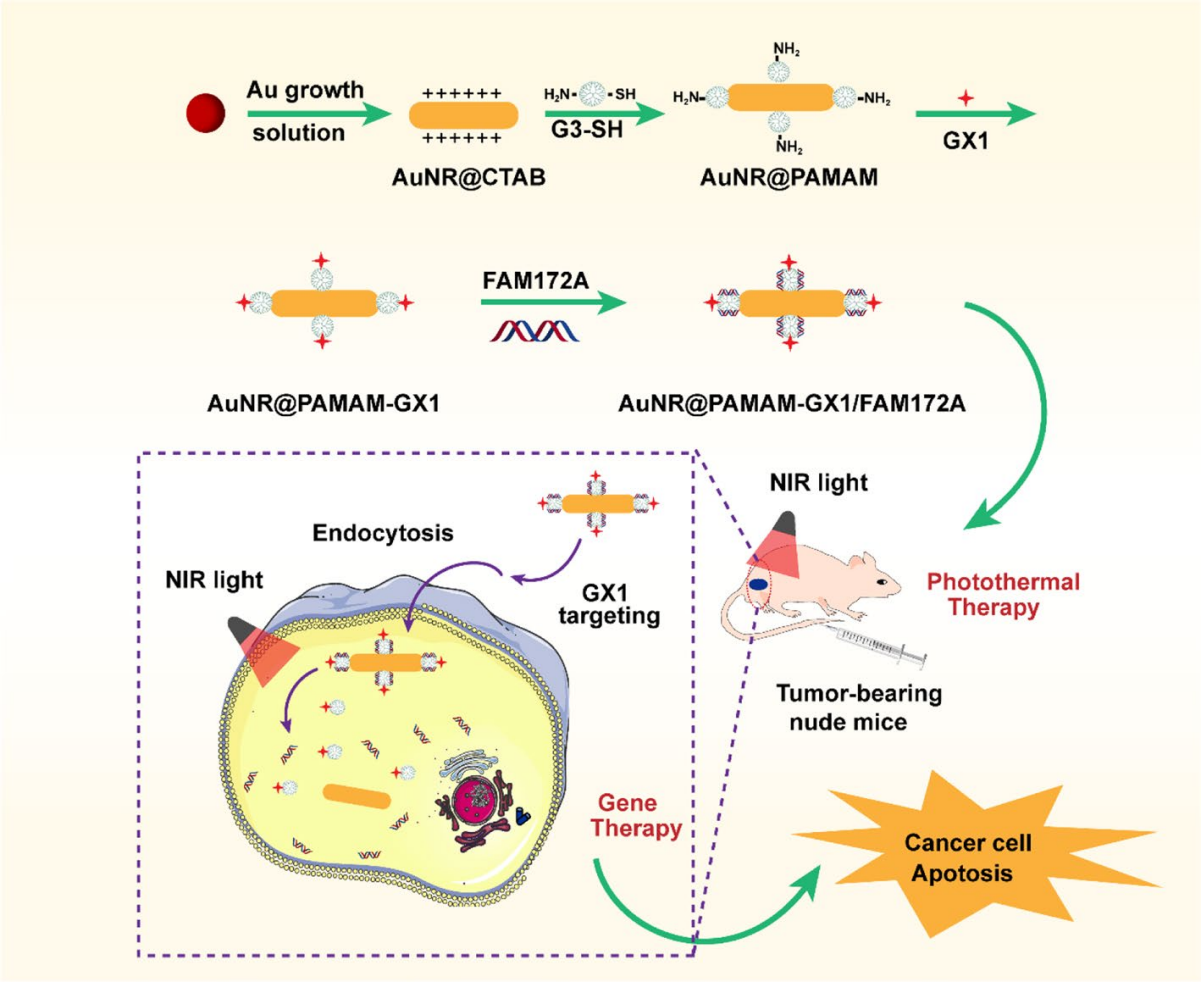
Schematic illustration of the GX1 targeting combinational gene therapy and photothermal therapy nanoparticles for colon cancers treatment.

Based on the above considerations, there is need for the development of a unique multi-functional nanoplatform, which can be used for imaging guided tumor combination therapy (Scheme 1). The obtained Au NR@PAMAM-GX1/FAM172A complex had good function of CT imaging and photothermal efficacy. In vitro experiments illustrated that Au NR@PAMAM-GX1/FAM172A inhibit cancer colon cancer cells growth and proliferation and induce apoptosis. Further, the results of animal experiment studies demonstrated that Au NR@PAMAM-GX1/FAM172A had excellent tumor ablation therapeutic effect and superior anti-tumor efficacy.

## Materials and methods

### Materials

Cetyltrimethylammonium bromide (CTAB), chloroauric acid (HAuCl_4_), sodium borohydride (NaBH_4_), silver nitrate (AgNO_3_), L-ascorbic acid, methyl 2-sulfanylacetate, ethylenediamine and methyl acrylate were obtained from Aladdin Chemical Company (Shanghai, China). N-hydroxysuccinimide (NHS) and 1-(3-dimethylaminopropyl)-3-ethylcarbodiimide (EDC) were obtained from Shanghai Yuanye Bio-Technology Co., Ltd. (Shanghai, China). GX1 peptide was bought from Apeptide Bio-Technology Co., Ltd. (Shanghai, China). FAM172A were from RiboBio Co., Ltd. (Guangzhou, China). All the other chemical reagents used as received from the suppliers without further purification.

### Synthesis of nanoparticles

#### Synthesis of PAMAM-G_3_

PAMAM dendrimers were synthesized using the divergence method according to the literature report with slight modification [25]. Briefly, 50 mL of methanol was placed into a round-bottom flask equipped with a magnetic stirrer, reflux condenser, and thermometer. Ethylenediamine was dissolved in anhydrous methanol, dripped with excess methyl acrylate in methanol solution, stirred continuously for 48 h. Then, the solvent and monomer were separated by vacuum distillation to obtain PAMAM-G_0.5_ dendrimer. After stirring for 48 h, the PAMAM-G_0.5_ dendrimer was obtained by distillation under reduced pressure in anhydrous methanol, and then added dropwise to an excess of ethylenediamine in methanol solution at a rate of ~ 1 drop per second to obtain a PAMAM-G_1.0_ dendrimer. PAMAM-G_3.0_ can be obtained by repeating the above steps twice.

#### Synthesis of au NR

GNRs with long-wavelength LSPR peaks at 808 nm were synthesized in an aqueous solution using the seed-mediated template-assisted protocol [26]. Briefly, 0.6 mL of 10 mM ice-cold NaBH_4_ was injected into a 10 mL aqueous solution containing 0.1 M CTAB and 0.25 mM HAuCl_4_ under vigorous stirring. Then, 0.2 mL of 25 M HAuCl_4_ was added to 10 mL of 0.1 M CTAB to prepare the GNR growth solution. Subsequently, 40 μL of AgNO_3_ (16 mM) and 90 μL of ascorbic acid (80 mM) were added to the solution, respectively. After shaking, the growth liquid became colorless, and 12 μL of the previous gold seed solution was injected the growth solution. The mixture was allowed to stand at 37 °C for 12 h to promote GNRs growth. The synthesized GNRs were purified by centrifugation twice at 8000 rpm for 10 min, and then re-dispersed in deionized water.

#### Synthesis of au NR@PAMAM

A partially thiolated G_3_ (G_3_-SH) was synthesized, according to previous reports [27]. Briefly, the aqueous solution of G_3_-SH (20 mg in 1 mL of water) and Au NSs (10 mL) were mixed by ultrasonic treatment for 15 min, and then stirred at room temperature for another 24 h. The purified Au NR@PAMAM were obtained by centrifugation three times at 8000 rpm for 10 min and dispersed in DI water.

#### Synthesis of au NR@PAMAM-GX1

1.6 M equivalents of EDC and NHS were added to 1 mL of GX1 (20 mg/mL, DMSO) solution and stirred for 4 h. Then, the above solution was added to the Au NR@PAMAM dispersion under continuous stirring overnight. Finally, the purified Au NR@PAMAM-GX1 were obtained by centrifugation three times at 8000 rpm for 10 min and resuspended in DI water.

### Characterization

The chemical structures of PAMAM and PAMAM-SH was confirmed by ^1^H NMR spectroscopy (300 MHz, Varian, USA) using deuterium oxide (D_2_O) as a solvent. The morphology of Au NR@CTAB was observed using transmission electron microscopy (JEOL TEM-1210) at 120 kV. The zeta potential and particle size were measured with a Nano-ZS instrument (Malvern Instru-ments Limited, England). UV-Vis spectra of PAMAM-SH, Au NR@CTBA and Au NR@PAMAM were determined by using an Ultramicro ultraviolet spectrophotometer (UV-2550, Shimadzu Corporation, Japan), and full wavelength scanning was performed at 200–900 nm. The Fourier transform infrared (FTIR) spectra of all samples were recorded in a Fourier transform-infrared spectrometer (Vertex-70, Bruker, Germany). The component analysis of Au NR@PAMAM and AuNR@PAMAM-GX1 was determined using thermogravimetric analyses (TGA-50, Shimadzu, Japan).

### Preparation of au NR@PAMAM-GX1/FAM172A complex

To obtain Au NR@PAMAM-GX1/ FAM172A complexes, 50 μL of Au NR@PAMAM-GX1 solution and 50 μL of FAM172A at 4 different concentrations of N/P (where N = molar number of primary amines in the PAMAM and P = molar number of phosphate groups in FAM172A backbone). N/*P* = 15: 1, 20: 1, 30: 1, 40: 1 and 50: 1 were shaken followed by incubating at room temperature for 30 min. The Au NR@PAMAM-GX1/FAM172A complexes preparation was based on self-assembly due to electrostatic forces.

### Gel retardation assay

The FAM172A condensing ability of Au NR@PAMAM-GX1 was examined by gel retardation electrophoresis assay. Briefly, the AuNR@PAMAM-GX1/FAM172A complexes with different N/P ratios were separated by 1% agarose gel electrophoresis containing Gold View II nucleic acid stain (SBS Genetech Corp. Ltd., Beijing, China) at 150 V for 15 min. Gel images were acquired using a gel imaging analysis system (Bio-Rad, Hercules, CA, USA).

### Complex size and potential

The hydrodynamic sizes and zeta potentials of Au NR@PAMAM-GX1/FAM172A complexes were obtained using Zeta-Sizer instrument (Nano-ZS; Malvern Instruments, UK). Results were recorded in triplicate at least three independent experiments.

### Photothermal property of the AuNR@PAMAM-GX1

The photothermal property and stability of the Au NR@PAMAM-GX1 were examined. The aqueous suspension of Au NR@PAMAM-GX1 solution with different Au concentrations (10, 20 and 40 μg/mL) were monitored by continuously irradiating the materials in a tube filled with 0.5 mL water using an 808 nm laser (Changchun Lei Rui Optoelectronics Technology Co., Ltd., Jilan, China) at a power density of 1.5 W/cm^2^for 300 s. The temperature of different samples was recorded using a thermocouple probe every 10 s. The thermal images were captured with Fotric 226 infrared thermal imaging camera (Zmtong Technology Co., Ltd., Shenzhen, China). Next, the Au NR@PAMAM-GX1 with 40 μg/mL concentration of Au was irradiated by near-infrared laser for 300 s, with the laser density set at 0.5, 1, 1.5 and 2.0 W/cm^2^. Meanwhile, the Au NR@PAMAM-GX1 with 40 μg/mL concentration was irradiated by near-infrared laser (1.5 W/cm^2^) for 300 s, and the solution was cooled down to room temperature for 300 s. The irradiation and cooling process was carried out four times.

### CT image

X-ray attenuation property of Au NR@PAMAM-GX1 was performed using a LightSpeed VCT imaging system (GE Medical Systems, Milwaukee, WI, USA) with the parameters of 100 Kv, 80 mA, and a thickness of 0.625 mm. Au NR@PAMAM-GX1 dispersion with series of Au concentration was placed in 0.2 mL Eppendorf tubes for CT imaging. All images were converted to Hounsfield units (HU) through using the built-in software.

### Cell culture

Human colon adenocarcinoma cells (HCT-8 cells) and mouse fibroblast L929 cells (L929 cells) were all purchased from Beogene Biotechnology Co., Ltd. (Guangzhou, China). HCT-8 cells and L929 cells were cultured in DMEM midum were supplemented with 10% FBS and 1% double antibodies (100 mol/mL penicillin, 100 μg/mL streptomycin) at 37 °C in a 5% CO_2_ humidified incubator.

### Cytotoxicity of au NR@PAMAM-GX1

Cytotoxicity of Au NR@PAMAM-GX1 was determined using CCK-8 as previously reported. HCT-8 cells and L929 cells were plated in a 96-well plate with the concentration of 1 × 10^4^ cell/well. After 12 h of incubation, cells were treated with 100 μL of fresh cell medium containing Au NR@PAMAM-GX1 at different concentrations (10, 20, 40, 80 and 100 μg/mL), respectively. HCT-8 and L929 cells treated with fresh cell medium were used as control. The cells were maintained for 24 h, and then the medium was replaced by 100 μL of DMEM medium containing 10% CCK-8 solution and incubated for 1.5 h at 37 °C. Subsequently, 10 μL of CCK-8 was added to each well containing 100 μl culture medium, and the cells were cultured for 2 h. Finally, the absorbance in each well was determined with a microplate reader (MultiskanMk3, Sunnyvale, CA) at 450 nm and defined using the formula:

$$\mathrm{Cell}\ \mathrm{viability}\ \left(\%\right)=\frac{{\mathrm{A}}_{\mathrm{p}}-{\mathrm{A}}_{\mathrm{s}}}{{\mathrm{A}}_{\mathrm{p}}-{A}_{\mathrm{b}}}$$

Where, A_p_, A_s_, A_b_ are the absorbance of the positive control, sample and blank control, respectively.

### PTT and gene therapy in vitro

The photothermal ablation effect of Au NR@PAMAM-GX1 to tumor cells was evaluated with the HCT-8 cells in vitro. Briefly, HCT-8 cells were plated in a 96-well plate with the concentration of 1 × 10^4^ cell/well for 12 h. Thereafter, cells were treated with Au NR@PAMAM-GX1 at different Au concentrations for 24 h. The cells were washed with phosphate buffer three times, treated with 100 μL fresh medium and irradiated by near-infrared laser (808 nm, 1 W/cm^2^) for 5 min. Finally, cell viability was measured by the CCK-8 assay.

The therapy effect of PTT combined with gene to tumor cells was evaluated y CCK-8 assay. Cells were treated with Au NR@PAMAM-GX1 or Au NR@PAMAM-GX1/FAM172A polyplexes (N/*P* = 40: 1, 1 μg FAM172A) for 24 h, respectively. Cells were washed with phosphate buffer for three times, incubated with 100 μL FBS-free medium and irradiated by near-infrared laser (808 nm, 1 W/cm^2^) for 5 min. Finally, cell viability was measured by CCK-8 assay. Meanwhile, cells were stained with Calcein AM, and observed by fluorescence microscope (Zeiss, Jena, German).

### Apoptosis assay

HCT-8 cells were plated in a 24-well plate with the concentration of 5 × 10^4^ cell/well for 12 h. Thereafter, cells were treated with RPMI 1640 medium containing Au NR@PAMAM-GX1 or Au NR@PAMAM-GX1/FAM172A complexes with N/P ratio of 40. After 6 h of incubation, cells irradiated by near-infrared laser (808 nm, 1 W/cm2) for 5 min. Cells treated with fresh cell medium and only treated with laser irradiation or Au NR@PAMAM-GX1 were used as control. After 18 h of incubation, cells were digested by trypsinized and resuspended in 200 μL phosphate buffer. Then, cells were stained with 5 μl annexin V-PE and 5 μl 7-aminoactinomycin D (7-AAD) and incubated for 15 min at room temperature in the dark. Finally, the apoptotic cells were analyzed using a flow cytometry (Accuri C6, BD, USA).

### Cellular uptake assay

Fluorescein isothiocyanate (FITC) was used as a hydrophilic probe to label the Au NR@PAMAM-GX1 (red fluorescence). HCT-8 cells were seeded in a 2 cm confocal microscopy dish with the concentration of 2 × 10^5^ cell/well and incubated for 12 h. Thereafter, cells were treated with FITC-labeled Au NR@PAMAM-GX1 and incubated for a different time (1 h, 3 h and 6 h), respectively. The cells were washed with PBS twice and then fixed by 4% paraformaldehyde. The cellular nuclei (blue fluorescence) were stained with DAPI. The fluorescence images of the cells were captured by confocal laser scanning microscopy (FV 3000, Olympus, Japan).

### Targeting ability assay

The cellular uptake of AuNR@PAMAM-GX1 complex before and after GX1 functionalization was analyzed to confirm the targeting ability of GX1. Briefly, HCT-8 cells were plated in a 24-well plate with the concentration of 5 × 10^4^ cell/well at 37 °C in a 5% CO_2_ humidified incubator for 12 h. Thereafter, cells were treated with FITC-labeled Au NR@PAMAM-GX1 and incubated for a different time (1 h, 3 h and 6 h), respectively. Cells were washed with phosphate buffer, digested with trypsin, centrifuged at 2000 rpm for 5 min and suspended in 200 μ L phosphate buffer. Finally, the samples were detected by flow cytometry (Accuri C6, BD, USA), and the corresponding fluorescence intensity was quantified by FlowJo7.6.1 software.

### Cell transfection efficacy screenings

HCT-8 cells were applied to analyze in vitro gene transfection. Briefly, HCT-8 cells were plated in a 24-well plate with the concentration of 5 × 10^4^ cell/well for 12 h. In this study, pDNA is a non-functional gene with GFP fluorescence fragment, which was used to evaluate gene expression efficiency. Thereafter, cells were treated with fresh media containing Au NR@PAMAM-GX1/pDNA complexes with different ratios (N/P ratio = 15: 1, 20: 1, 30: 1, 40: 1 and 50: 1). After 24 h of incubation, the fluorescence images of the cells were collected using a fluorescence microscope (Zeiss, Jena, German) to test green fluorescent protein (GFP) expression. After culturing with different complexes for 2 h, cells were digested by trypsinized and resuspended in 500 μL PBS solution. The transfection efficiency was determined using a flow cytometry (Accuri C6, BD, USA). Cells incubated with PBS and Au NR@PAMAM-GX1 were used as the negative control, whereas PEI-25 k/pDNA as the positive control.

### In vivo infrared thermal imaging studies

All animal experiments approved by the Institutional Animal Care and Use Committee (IACUC) of the Zhujiang Hospital of Southern Medical University. In vivo infrared thermal imaging was captured using Fotric 226 infrared thermal imaging camera. HCT-8 tumor-bearing nude mice were anesthetized with 4% trichloroacetaldehyde hydrate (100 μL/10 g) while maintaining normal vital signs. When the tumor volume reached about 100 mm^3^, 100 μL of Au NR@PAMAM or 100 μL of Au NR@PAMAM-GX1 or 100 μL of PBS was then injected into the tumor-bearing mice through tail intravenous injection. Six hours after injection, the mice were irradiated with near-infrared laser (1 W/cm^2^) for 300 s, and the thermal image and body temperature distribution of the mice were recorded using an infrared thermal imager. To evaluate the CT imaging effect of nanoparticles in vivo, the tumor-bearing nude mice were anesthetized and injected with Au NR@PAMAM and Au NR@PAMAM-GX1. Next, the mice were imaged by a 64-slice CT system (LightSpeed VCT; GE Healthcare, USA).

### Tumor inhibition assay

HCT-8 tumor-bearing nude mice were randomly assigned to five groups with 3 mice per group: PBS (injected with PBS), NIR (injected with PBS and irradiated by NIR laser), Au NR@PAMAM-GX1 ([Au] = 0.5 mg/kg), Au NR@PAMAM-GX1 + NIR (injected with Au NR@PAMAM-GX1 and irradiated by NIR laser, [Au] = 0.5 mg/kg), Au NR@PAMAM-GX1/FAM172A (injected with injected with Au NR@PAMAM-GX1/FAM172, [Au] = 0.5 mg/kg FAM172 = 10 mg/kg) and Au NR@PAMAM-GX1/FAM172 + NIR (injected with Au NR@PAMAM-GX1/FAM172A and irradiated by NIR laser, [Au] = 0.5 mg/kg, FAM172 = 10 mg/kg). Herein, the NIR means light irradiation by near-infrared laser (1 W/cm^2^) for 300 s. Treatment was conducted every 2 days for 14 days. Meanwhile, the size of the tumors was measured using an electronic caliper. The volumes of the tumors were calculated as 1/2 × shortest diameter^2^ × longest diameter and the weight of mice was recorded. After 14 days of feeding, the tumors were photographed and weighted.

### Histologic and immunohistochemical analysis

The mice of all groups was sacrificed at days 14 post-treatment. For histological analysis, the tumors were removed, embedded in paraffin. The embedded blocks were cut into 4-μm-thick sections and stained with haematoxylin and eosin (H&E). For immunohistochemical analysis, the level of tumor apoptosis was examined using the terminal deoxynucleotidyl transferase deoxyuridine triphosphate nick-end labeling (TUNEL) assay. Meanwhile, the expression of Ki67 in tumor sections was evaluated using immunohistochemical analysis. Western blot analysis of FAM172A at tumor tissue was performed according to standard protocols.

### Statistical analysis

All quantitative data were shown as mean ± standard deviation. Statistical comparisons were performed using One-way ANOVA. Significant difference: * (p<0.05), ** (p<0.01), and *** (p<0.001).

## Results and discussion

### Synthesis and characterization of the au NR@PAMAM-GX1

CTAB-coated Au NRs were synthesized by a seed-mediated growth method. Partially thiolated G3 PAMAM dendrimers was completed by reacting G3.0 PAMAM dendrimers with methyl mercapto acetate. The resultant Au NRs were conjugated with thiolated G3.0 dendrimers via Au-S bond formation. The Au NR@PAMAM-GX1 was prepared by GX1 with Au NR@PAMAM. The Au NR@PAMAM-GX1 was combined with the FAM172A for CT/thermos imaging and the combination of PTT and gene therapy of tumors.

**Fig. 1:**
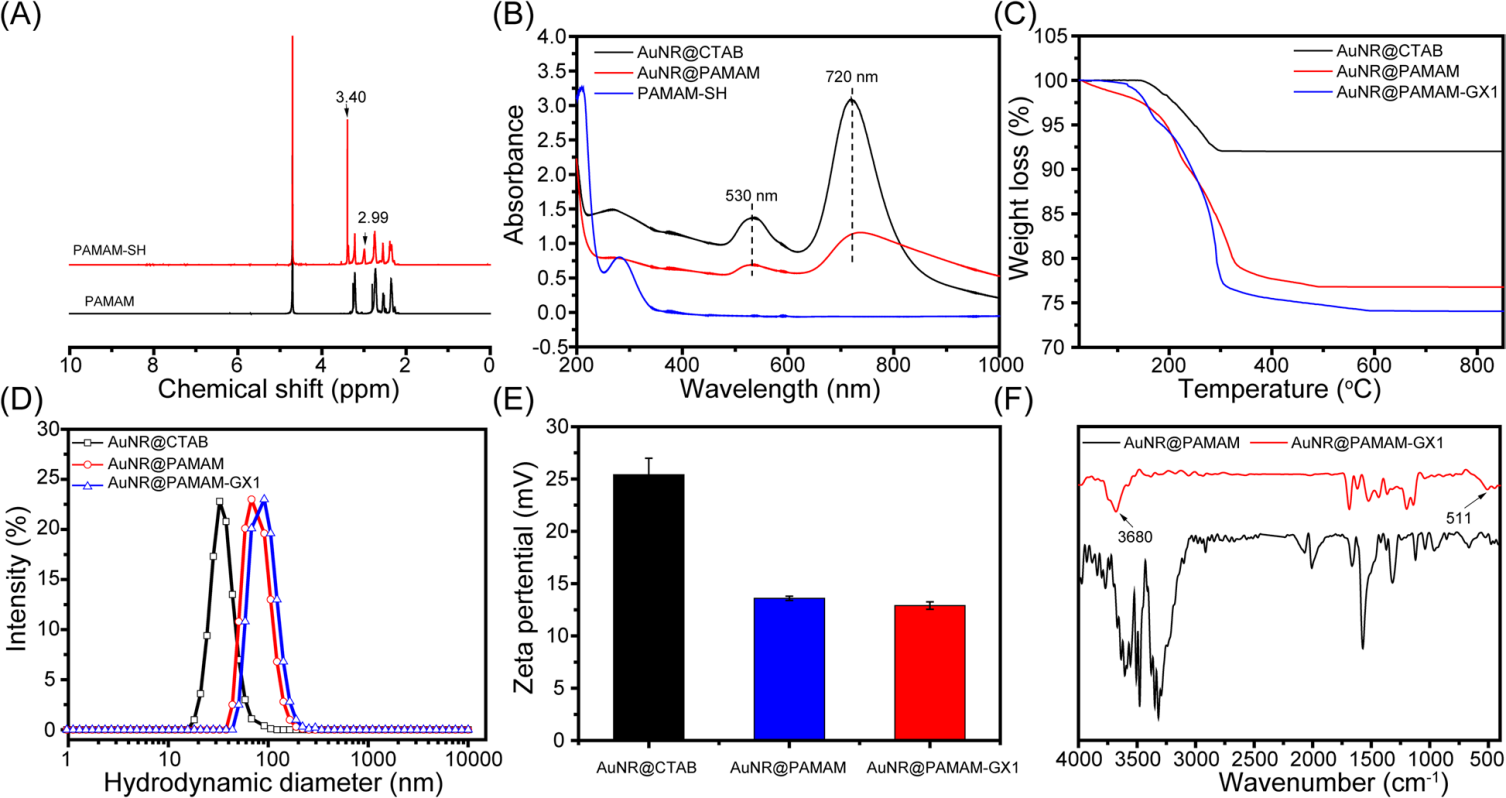
Characterizations of AuNR@PAMAM-GX1. **A** ^1^H NMR spectra for PAMAM and PAMAM-SH. **B** UV-Vis spectra of Au NR@CTAB, AuNR@PAMAM and PAMAM-SH. **C** TG curves of Au NR@CTAB, Au NR@PAMAM and Au NR@PAMAM-GX1. **D** Hydrodynamic diameter of Au NR@CTAB, Au NR@PAMAM and Au NR@PAMAM-GX1. **E** Zeta potential of Au NR@CTAB, Au NR@PAMAM and Au NR@PAMAM-GX1. **F** FTIR spectra of Au NR@PAMAM and Au NR@PAMAM-GX1

The G3-SH was first investigated using ^1^H NMR spectroscopy (Fig. 1A). Compared with amine-terminated G3, G3-SH showed an additional peak at 3.40 ppm, which could be assigned to the characteristic methylene peak of -NHCO-CH_2_-SH. Transmission electron microscopy (TEM) images indicated that the formed Au NRs have a nice rod shape with an average length of ∼50 nm and an average width of ∼20 nm (Fig. S1). The formation of the Au NR@CTAB was also confirmed with the diffuse reflectance UV–visible absorbance method. As shown in Fig. 1B, Au NR@CTAB has obvious absorption characteristics in the near-infrared region. However, the formed Au NR@PAMAM-GX1 has a clear surface plasmon resonance (SPR) peak at 720 nm, which shows great potential in the applications on PTT. Meanwhile, compared with Au NR@CTAB before dendrimer grafting, dendrimer plus GX1 peptide modification to Au NR does not cause a significant change in SPR. As shown in Fig. 1C, TG measurement quantified the composition of the Au NR@PAMAM-GX1, in which Au, PAMAM dendrites and GX1 accounted for 22.4%,74.04 and 3.56% of the entire nanosystem, respectively. As shown in Fig. 1D, Au NR@PAMAM and Au NR@PAMAM-GX1 have a narrower size distribution with hydrodynamic diameter (D_h_) of 74 nm and 102 nm, respectively. As shown in Fig. 1E, the surface charge of Au NR@PAMAM appears to decrease slightly owing to the GX1 combination, but the total charge of Au NR@PAMAM-GX1 was still positive. The solubility and stability were essential factor for the biomedical application of nanomaterials. As shown in Fig. 1F, FTIR shows that Au NR@PAMAM-GX1 has a typical disulfide bond absorption peak at 511 cm^− 1^.

### Photothermal property of the au NR@PAMAM-GX1

**Fig. 2:**
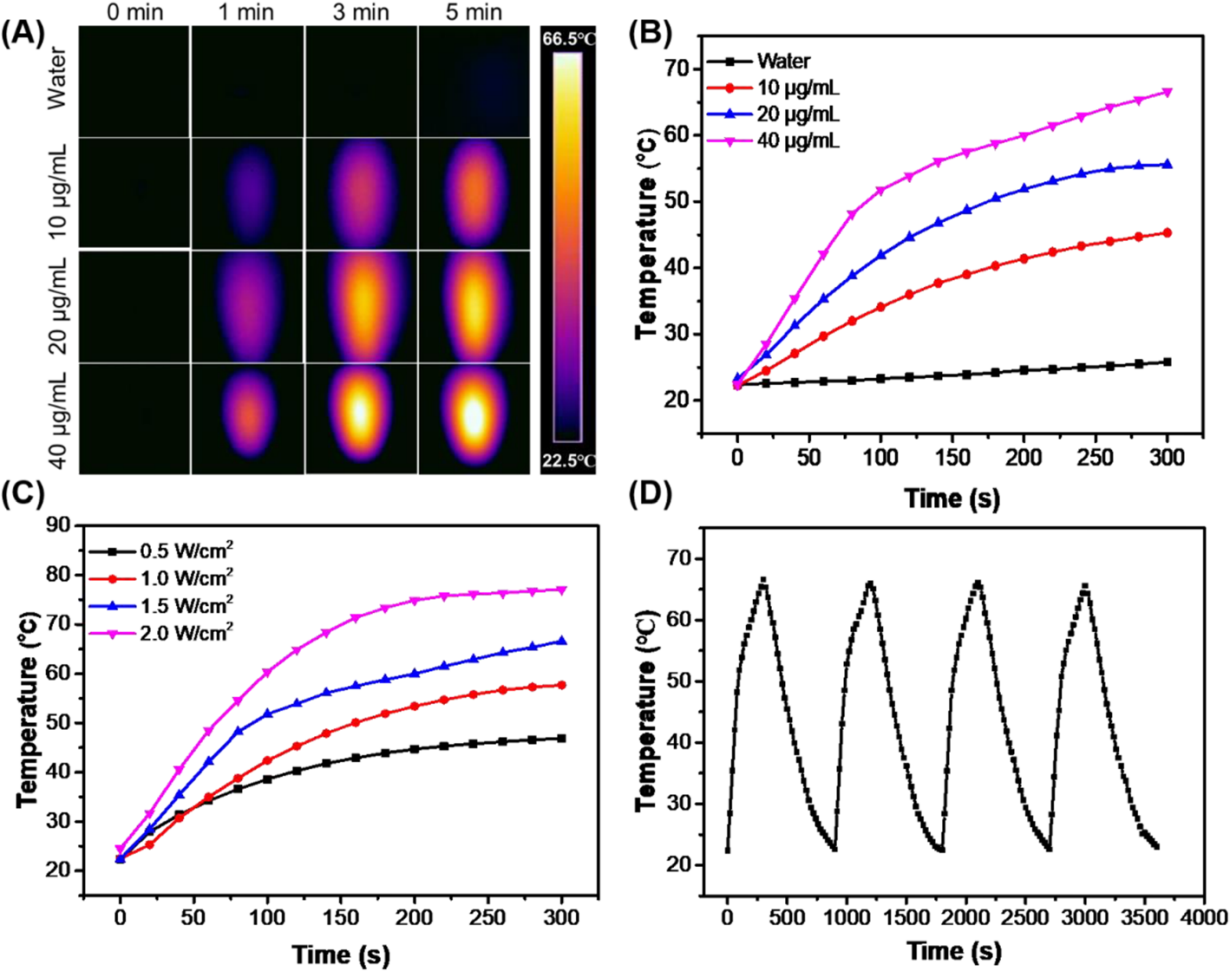
**A** Infrared thermal images and **B** Temperature of the solution containing Au NR@PAMAM-GX1 under the NIR laser (808 nm, 1.5 W/cm^2^) irradiation for 5 min. **C** Temperature of the solution containing Au NR@PAMAM -GX1 at different laser power density. **D** Temperature of the solution containing Au NR@PAMAM-GX1 (40 μg/mL) under laser ON/OFF cycles of NIR laser (1.5 W/cm^2^) irradiation

Gold nanorods have strong absorbance in the near infrared region, which have been widely studied and applied in photothermal therapy in vitro and in vivo**.** The photothermal property of the Au NR@PAMAM-GX1 was evaluated by laser t 808 nm. The photothermal heating curve tested by the infrared thermal camera showed strong concentration-dependent effects (Fig. 2A and B) and laser power-dependent effects (Fig. 2C), with a maximum temperature increment of 43 °C. However, the temperature of pure water does not have an obvious increase even under exposure to a high laser power density of 1.5 W/cm^2^. Meanwhile, the photothermal stability of Au NR@PAMAM-GX1 was tested by five cycles of irradiation (laser on, 808 nm, 1.5 W/cm^2^, 5 min) and a natural cooling process without NIR laser irradiation (laser off, 5 min) (Fig. 2D). In addition, no obvious change was observed in the maximum temperature value of Au NR@PAMAM-GX1, which indicated that the Au NR@PAMAM-GX1 is expected to be used as PTT agents for tumor treatment [28].

### X-ray attenuation property of the au NR@PAMAM-GX1

**Fig. 3:**
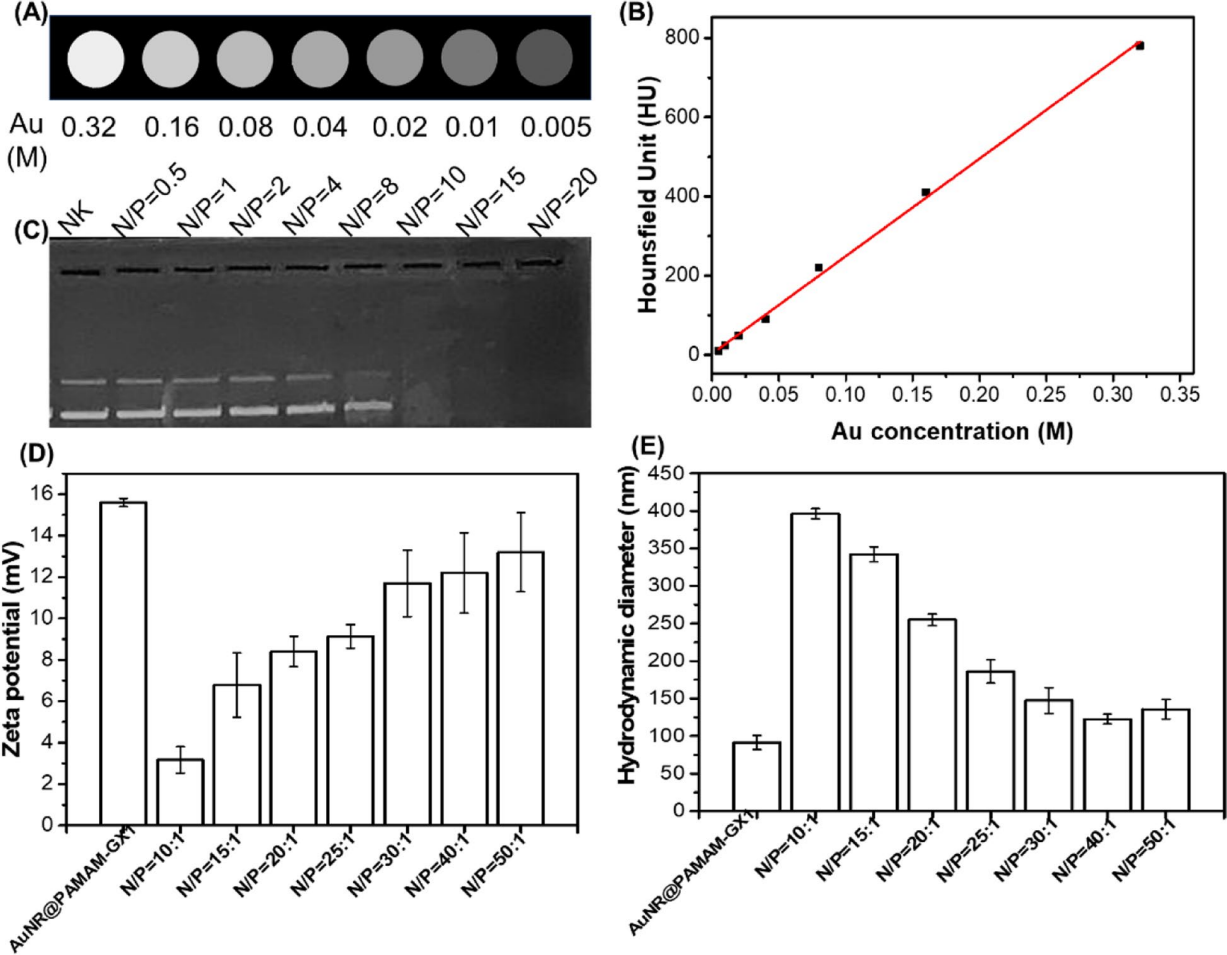
Characterization of Au NR@PAMAM-GX1 and Au NR@PAMAM-GX1/FAM172A complexes. CT images (**A**) and X-ray attenuation intensity (**B**) of Au NR@PAMAM-GX1 with different concentrations. **C** Gel retardation assay of Au NR@PAMAM-GX1/FAM172A with different N/P ratios. **D** Zeta potential and (**E**) hydrodynamic diameter of Au NR@PAMAM-GX1/FAM172A with different N/P ratios

Due to the high atomic number, gold nanoparticles have been studied as potential contrast agents for X-ray CT imaging [29]. Therefore, the potential of Au NR@PAMAM-GX1 as CT contrast agents was evaluated by X-ray attenuation intensity measurements. As shown in Fig. 3A, CT signal intensity continuously increases with the increasing of Au concentrations, resulting in brighter images. We can safely conclude that our AuNR@PamAM-GX1 is clinically promising as a CT imaging contrast agent, which is consistent with the previously reported literature. At an Au concentration of 0.04 M, the brightness of the CT image is significantly improved, which is consistent with the quantitative analysis of the relationship between the X-ray attenuation intensity (also known as the Hounsfield unit Hu) of AuNR@PAMAM-GX1 and the Au concentration (Fig. 3C). Thus, we can conclude that Au NR@PAMAM-GX1 may be a promising contrast agent for CT imaging in future clinical applications, which is consistent with the previously reported results [30].

### Formation and characterization of au NR@PAMAM-GX1/FAM172A complexes

Because naked FAM172A is easily degraded by nucleases during intracellular delivery, and negatively charged FAM172A is also difficult to pass through negatively charged cell membranes, we used Au NR@PAMAM-GX1 with positive surface potential as a carrier for delivery FAM172A. As shown in Fig. 3B, the gel retardation assay was performed to measure the gene compaction ability of the Au NR@PAMAM-GX1 through electrostatic interaction. The results showed that the migration of FAM172A with Au NR@PAMAM-GX1 at N/P ratio of 10 or more was completely retarded. Therefore, to form a stable complex between FAM172A and Au NR@PAMAM-GX1, an N/P ratio higher than 10 was selected. Next, the zeta potentials and sizes of the Au NR@PAMAM-GX1/FAM172A complexes with weight ratio ranging from 10: 1 to 50: 1 were measured. As shown in Fig. 3E, with the increasing of the N/P ratio, the size of the Au NR@PAMAM-GX1/FAM172A complexes gradually decreased. As shown in Fig. 3D, the zeta potential of all the constructed complexes was reduced under each studied N/P ratio compared to the individual carriers, which is due to the charge shielding effect of negatively charged FAM172A. When the N/P ratio is 40:1 or higher, the size and zeta potential values return to the level of Au NR@PAMAM-GX1, indicating that their stability is restored. These results indicate that Au NR@PAMAM-GX1 could become dense complexes with FAM172A, and their positive charge and particle size contribute to effective endocytosis.

### Cytotoxicity assay

**Fig. 4:**
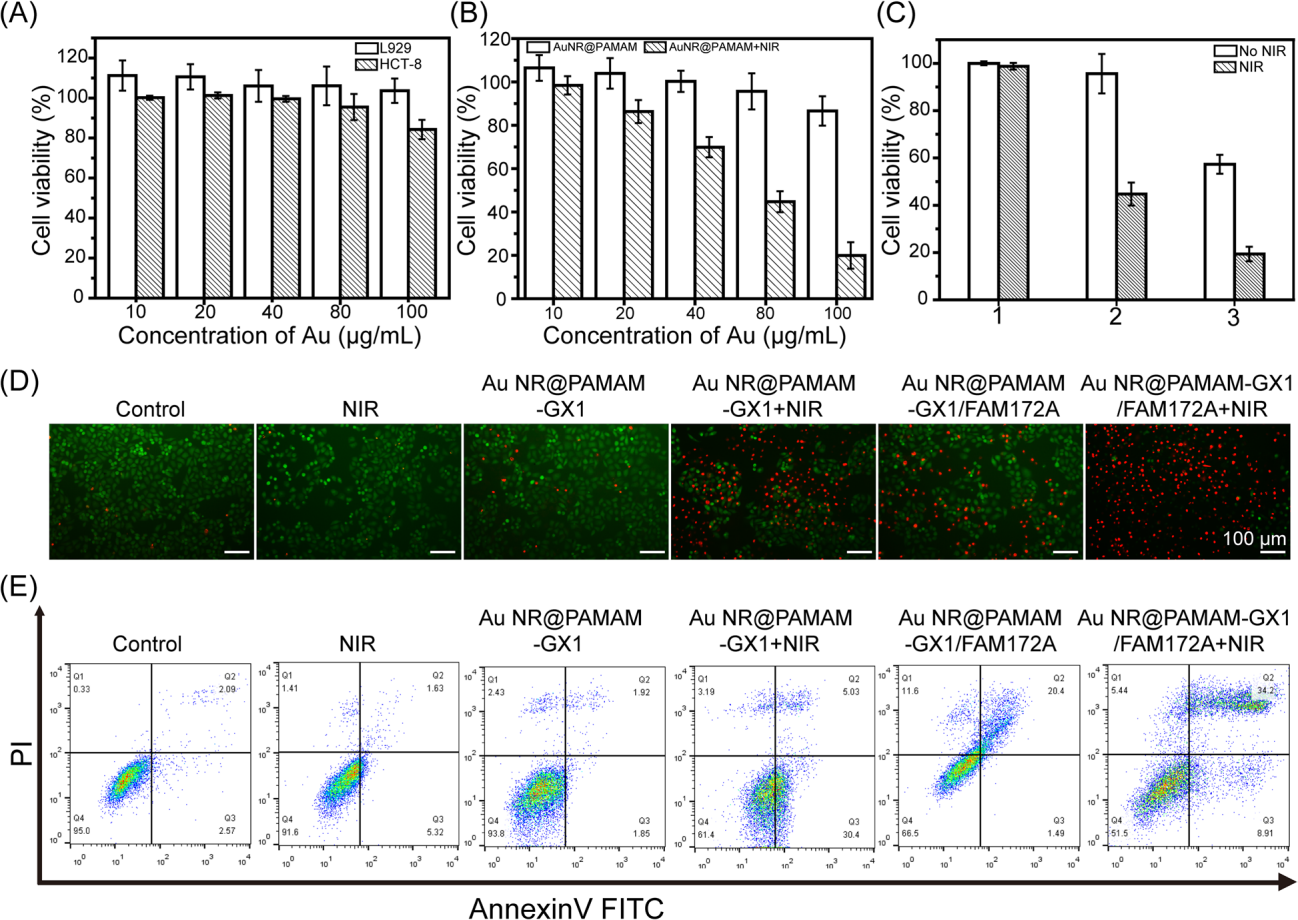
**A** In vitro cytotoxicity of Au NR@PAMAM-GX1 on HCT-8 cells and L929 cells over an evaluation period of 24 h. **B** Cell viability of HCT-8 cells pretreated by Au NR@PAMAM-GX1 with different concentrations with and without NIR irradiation. **C** CCK-8 assay of HCT-8 cells viability after treatment with the Au NR@PAMAM-GX1or Au NR@PAMAM -GX1/FAM172A polyplexes (N/*P* = 40: 1, 1 μg FAM172A per well) for 24 h, followed by laser irradiation for 5 min (1: PBS; 2: Au NR@PAMAM-GX1; 3: Au NR@PAMAM-GX1/FAM172A). **D** Images of PI and Calcein-AM double-stained HCT-8 cells under different treatments. **E** Flow cytometry analysis of HCT-8 cell apoptosis in different treatment groups

Before in vitro gene transfection evaluation, the cytotoxicity of Au NR@PAMAM-GX1 on HCT-8 cells and L929 cells were evaluated using CCK-8 assay. As shown in Fig. 4A, the viability of HCT-8 cells treated with Au NR@PAMAM-GX1 gradually decreased with the increasing of Au concentration. At the Au concentration up to 500 μg/mL, the cell viability remained more than 80%, which indicates that the selection of concentration range is reasonable. The low cytotoxicity of Au NR@PAMAM-GX1 may result from the good biocompatibility of PAMAM and the biological inertness of Au nanoparticles.

### Combinational PTT and gene therapy of cancer cells in vitro

The good photothermal conversion efficiency of the Au NR@PAMAM-GX1 prompted us to use them for laser ablation of cancer cells in vitro. The survival rate of HCT-8 cells incubated with different Au concentrations of Au NR@PAMAM-GX1 under laser irradiation was measured by the CCK-8 method. As shown in Fig. 4B, HCT-8 cells treated with AuNR@PAMAM-GX1 at different Au concentrations (10, 20, 40, 80 and 100 μg/mL) without laser irradiation did not show any obvious changes in survivability. After incubated with Au NR@PAMAM-GX1 with laser irradiation for 5 min, the viability of HCT-8 cells gradually decreased with the increasing of Au concentration. When the Au concentration is 80 μg/mL, almost 55.3% of cells are killed. These results indicate that AuNR@PAMAM-GX1 has an excellent tumor destruction effect.

To explore the potential of Au NR@PAMAM-GX1 polymer in combination with PTT and gene therapy cancer cells in vitro, the viability of HCT-8 cells was evaluated by CCK-8 assay. As shown in Fig. 4C, the survival rate of HCT-8 cells treated with Au NR@PAMAM-GX1 with laser irradiation for 5 min was 44.73%, while the survival rate of HCT-8 cells treated with Au NR@PAMAM-GX1/FAM172A polyplexes without laser irradiation was 57.3%. However, after cultivated with Au NR@PAMAM-GX1/FAM172A polyplexes with laser irradiation for 5 min, the cell survival rate dropped to 19.3%, which highlighted the effects of PTT enhancement and supercell growth inhibition after gene therapy. Fluorescence microscopy imaging of cells after different treatments further confirmed the enhancement of the treatment effect. As shown in Fig. 4D, the number of living cells (green cells) in all control groups is Au NR@PAMAM-GX1/FAM172A + laser < Au NR@PAMAM-GX1 + laser < Au NR@PAMAM-GX1/FAM172A < all control groups. The results showed that the combination of photothermal and gene therapy was more effective in inhibiting cell proliferation.

### Cell apoptosis

In order to further evaluate the cytotoxicity and cell death induced by gene and PTT treatment of Au NR@PAMAM-GX1 nanocomposite materials, Annexin V-FITC and PI dye staining methods were used to detect cell apoptosis. As shown in Fig. 4E, most of the cells (95.35%) in the control group were still alive. However, under light conditions, in the groups treated with Au NR@PAMAM-GX1 and Au NR@PAMAM-GX1/F172A, the proportion of viable cells decreased significantly, and the apoptosis rates were 35.43 and 43.11%, respectively. These results indicate that the genes of Au NR@PAMAM-GX1/F172A nanocomposite and apoptosis induced by the PTT effect are the main causes of cell death.

### Cellular uptake of au NR@PAMAM-GX1

**Fig. 5:**
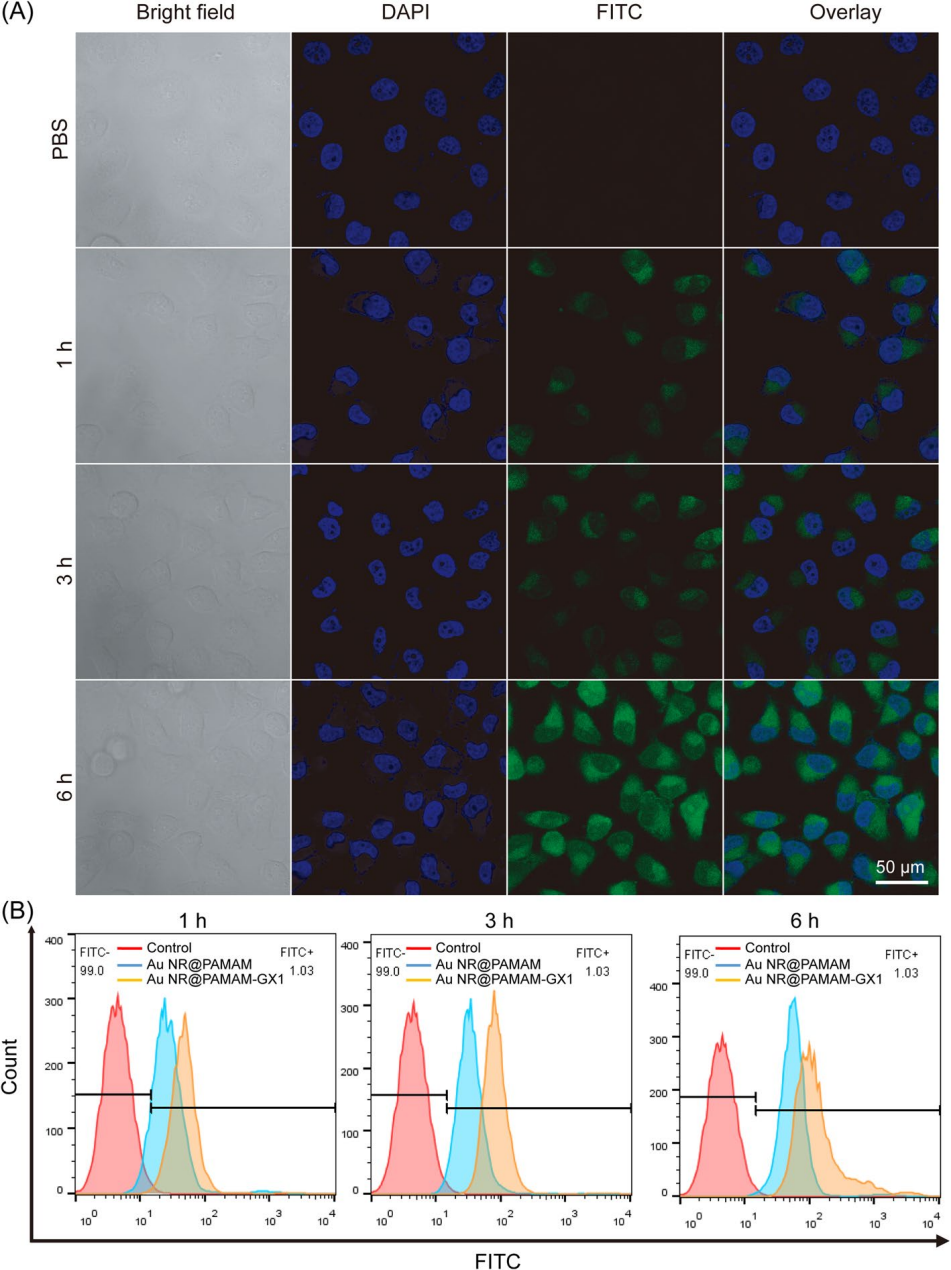
**A** Cellular uptake evaluation of Au NR@PAMAM-GX1 using CLSM over an incubation period of 6 h, where nuclei were stained by DAPI displaying blue fluorescence, Au NR@PAMAM-GX1 were FITC labeled showing green fluorescence. **B** Flow cytometry analysis of fluorescence peak Fig. in HCT-8 cells incubated with Au NR@PAMAM or Au NR@PAMAM-GX at different time

The efficient absorption of cells in vivo is the key to achieving the good biological performance of biomaterials [31]. Therefore, the uptake of Au NR@PAMAM-GX1 by HCT-8 cells was analyzed using a confocal laser scanning microscope (CLSM), in which Au NR@PAMAM-GX1 was fluorescently labeled with FITC. As shown in Fig. 5A, the internalization of Au NR@PAMAM-GX1 showed a time-dependent shift. With the increasing of fluorescence intensity, it indicated that more substances were internalized under a longer incubation period. Among them, Au NR@PAMAM-GX1 began to enter the cell at 1 h, mainly accumulating in the cytoplasm. Obviously, the cells treated with the complex for 6 h showed a stronger green fluorescent signal than other time points, which indicated that the most enriched materials in the cells were at the changed time point.

To prove the targeting ability of GX1 to HCT-8 cells, the cell uptake of Au NR@PAMAM and Au NR@PAMAM-GX1 was also determined by flow cytometry. As shown in Fig. 5B, Au NR@PAMAM with GX1 functionalization had the strongest fluorescence intensity at the same time point, indicating that Au NR@PAMAM-GX1 absorbed by HCT-8 cells increased significantly. As shown in Fig. S2, the quantitative analysis results indicated that the FITC intensity the Au NR@PAMAM-GX1 group was the highest. These results indicate that GX1 does improve the delivery efficiency of the nano-system to HCT-8 cells.

### In vitro gene transfection

**Fig. 6:**
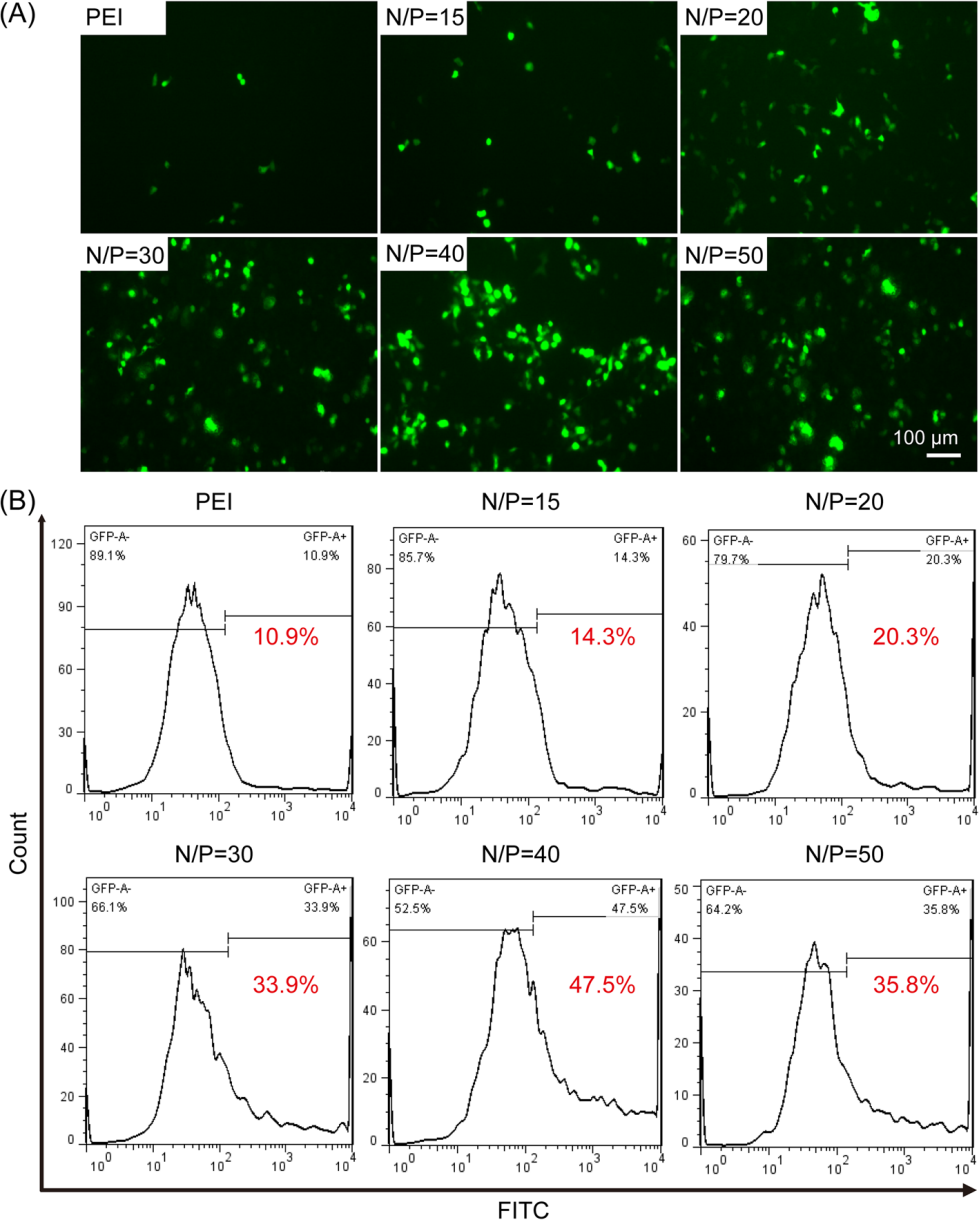
In vitro gene transfection efficiency evaluation of Au NR@PAMAM -GX1/pDNA complexes. **A** In the presence of serum, fluorescence images of HCT-8 cells transfected with Au NR@PAMAM-GX1/pDNA complexes in different weight ratios. **B** The gene transfection efficiency of Au NR@PAMAM-GX1/pDNA with different N/P ratios were determined using flow cytometry

The feasibility of Au NR@PAMAM-GX1 as gene transfection vectors was assessed by GFP gene expression experiments. The expression of GFP in HCT-8 cells under different complex treatments was evaluated by the fluorescence microscopy. As shown in Fig. 6A, compared with the PEI-25 k/pDNA complex, Au NR@PAMAM-GX1/pDNA complex at each N/P ratio treated HCT-8 cells had stronger fluorescence intensity, which indicated that pDNA delivery efficiency of Au NR@PAMAM-GX1 is higher than PEI-25 k. Moreover, the number of HCT-8 cells transfected with Au NR@PAMAM-GX1/pDNA complex with an N/P ratio of 40: 1 was observed to be the largest and the fluorescence intensity was the strongest, which was consistent with the results of flow cytometry experiments.

In addition, flow cytometry was used to detect the in vitro gene transfection efficiency of Au NR@PAMAM-GX1/pDNA complex with different N/P ratios in serum-containing medium. PEI-25 k/pDNA were set as control. As shown in Fig. 6B, the transfection efficiency of the Au NR@PAMAM-GX1/pDNA complex was dependent on the N/P ratio. Notably, the gene transfection efficiency of the Au NR@PAMAM-GX1/pDNA complex with an N/P ratio of 40: 1 is the highest, with a total of 47.5% of HCT-8 cells transfected. Meanwhile, the transfection efficiency of the Au NR@PAMAM-GX1/pDNA complex was significantly higher than that of PEI-25 k/pDNA complex, and only 10.9% of cells were transfected.

### In vivo thermal imaging and CT imaging

Taking advantage of the excellent photothermal properties of the Au NR@PAMAM-GX1, we explored the feasibility of using it for thermal imaging of xenograft tumor models. As shown in Fig. S3, the whole body thermal image of the mouse during the laser irradiation showed that the temperature of the tumor area after the injection of PBS only increased by 3.2 °C, while the temperature of the tumor area after the injection of Au NR@PAMAM and Au NR@PAMAM-GX1 showed significant temperature increases at 20.1 and 30.7 °C. Compared with Au NR@PAMAM, the temperature increase of using Au NR@PAMAM-GX1 is better, which may be due to the targeting effect of GX1 polypeptide that makes more materials enrich in tumor sites.

Previous results in vitro showed that Au NR@PAMAM-GX1 has excellent CT imaging effect, and the CT imaging effect of Au NR@PAMAM-GX1 on tumor sites in nude mice is further validated in vivo. As shown in Fig. S4A, it is difficult to clearly seen Au NR@PAMAM on tumor sites. However, the location of Au NR@PAMAM-GX1 on tumor sites can be clearly distinguished at 30 min post injection. As time passed, larger amounts of Au NR@PAMAM-GX1 were successfully enriched on tumor sites, which were due to that GX1-coated nanoparticles could target the tumor sites. Quantitative analysis of the brightness (HU value) of tumor tissue further demonstrated that the Hu value at 90 min after injection of Au NR@PAMAM-GX1 was significantly higher than Au NR@PAMAM (Fig. S4B). The Hu value increased from 25 to 180, which indicated that Au NR@PAMAM-GX1 has a good targeted imaging effect of CT in vivo.

### In vivo enhanced PTT and gene therapy of tumors

**Fig. 7:**
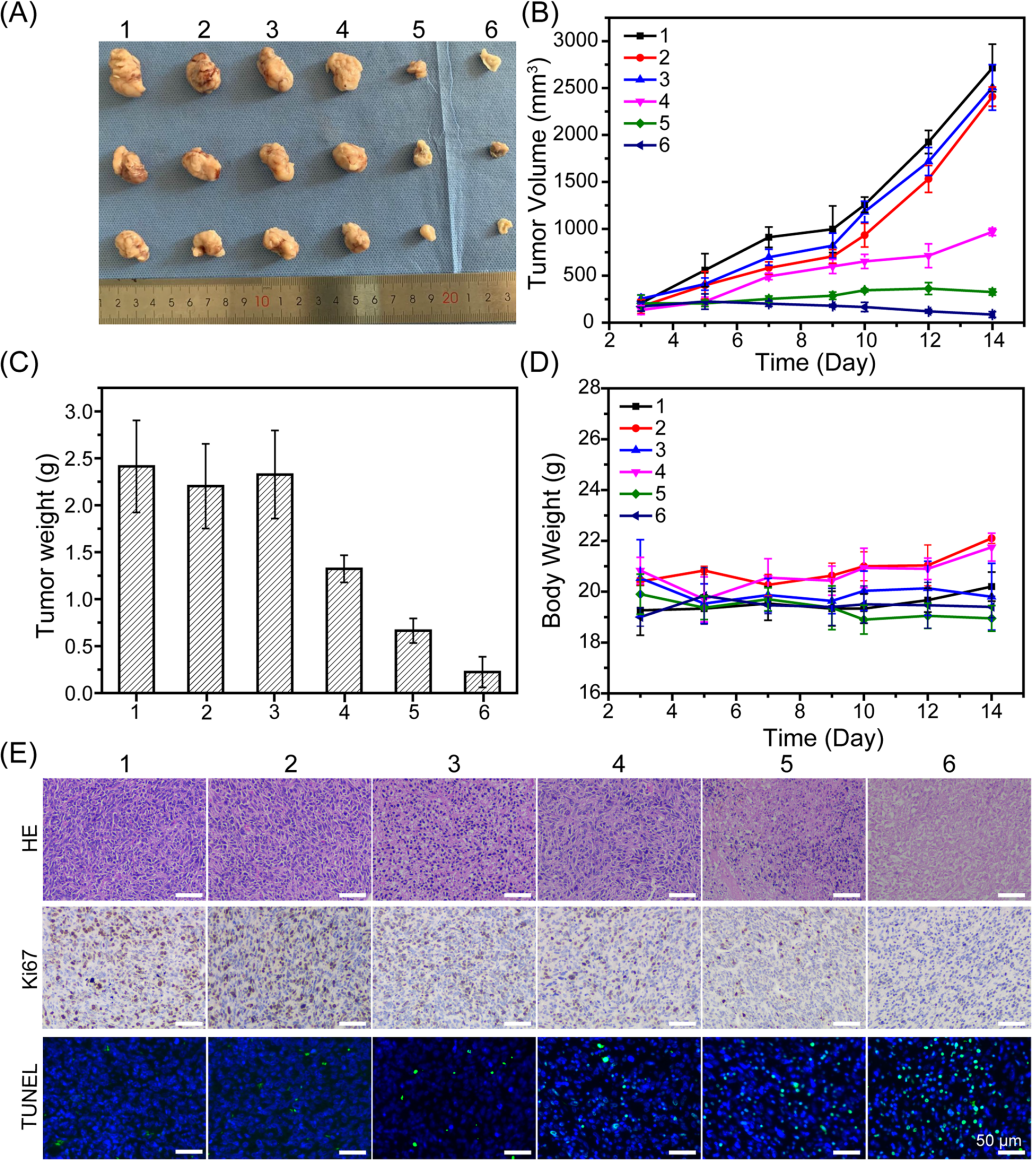
**A** In vivo tumor growth curves of HCT-8 tumor-bearing mice treated with different formulations. **B** Representative image of HCT-8 tumors at the 14st day. **C** The tumor weights excised from different groups after 14 days treatment. **D** Body weight changes of mice treated with different formulations during the treatment. **E** Immunohistochemical analyses of H&E, TUNEL, CD31 and Ki67 for HCT-8 tumor tissues after the last treatment with different formulations in vivo (400×) (1: PBS; 2: PBS + NIR; 3: Au NR@PAMAM-GX1; 4: Au NR@PAMAM-GX1 / FAM172A; 5: Au NR@PAMAM-GX1 + NIR; 6: Au NR@PAMAM-GX1/FAM172A + NIR)

We then measured the PTT and gene therapy effect of Au NR@PAMAM-GX1 HCT-8 tumor-bearing nude mice. The tumor suppression effects of different treatments were monitored by measuring tumor volume and tumor weight. As shown in Fig. 7A, the tumor growth curves of mice in different treatment groups were obvious within 14 days. Meanwhile, the relative tumor volume of the control group (with or without laser irradiation) and Au NR@PAMAM-GX1 without laser irradiation increased rapidly. However, the multiple clusters of Au NR@PAMAM GX1/FAM172A without laser irradiation reduced the tumor growth rate to a certain extent, which was may due to the role of gene therapy. In addition, the treatment of Au NR@PAMAM-GX1 and Au NR@PAMAM-GX1/FAM172A multi-complex under laser irradiation significantly inhibited tumor growth, which indicated that the combination of PTT and gene therapy can make tumor ablation most effective. As shown in Fig. 7B and C, it clearly shows that the anti-tumor effect of the material after the order Au NR@PAMAM-GX1/ FAM172A (NIR+) > Au NR@ PAMAM-GX1 (NIR+) > Au NR@PAMAM-GX1/ FAM172A (NIR−) > Au NR@PAMAM-GX1 (NIR−) > PBS control. As shown in Fig. 7D, there was no significant change in the body weight of the mice under different treatments at different time points, which indicated that the injection of Au NR@PAMAM-GX1 or Au NR@PAMAM-GX1/FAM172A material, regardless of whether the laser irradiation or not, will not affect the growth state of the mouse. All results indicate that the prepared material is not toxic to the mouse.

### Histologic and immunohistochemical analysis

The anti-tumor effect of Au NR@PAMAM-GX1/FAM172A was further evaluated by histopathological analysis of HCT-8 tumor sections stained with H&E. As shown in Fig. 7E and Fig. S5, the tumor cells treated with PBS and the single light group had a complete structure and more chromatin, indicating tumor was rapid growth. The other groups treated with Au NR@PAMAM-GX1/FAM172A showed tumor cell nuclear shrinkage of different degrees and enlarged intracellular space, which suggested that these groups had effective treatment responses to tumors. The tumors treated by Au NR@PAMAM-GX1/ FAM172A under light conditions have the largest intracellular space and the fewest tumor cells, which indicated that the combination therapy group showed the best therapeutic effect.

The terminal deoxynucleoside transfer-induced dUTP labeling terminal labeling (TUNEL) method was used to further evaluate the effects of different treatments on apoptosis in vivo. Similarly, Au NR@PAMAM-GX1/FAM172A + NIR induced the highest proportion of apoptosis-positive tumor cells, which confirmed that it has strongest anticancer activity in vivo. Tumor sections of each group were stained with Ki67 immunohistochemical staining to monitor changes in tumor cell proliferation activity. Ki67 is a sign of cell proliferation, in which light brown represent positive expression of Ki67 and blue indicates negative expression of Ki67. Ki67 staining results showed that Au NR@PAMAM-GX1/FAM172A + NIR treatment could significantly reduce Ki67-positive cells, which indicated that it could suppress the proliferation of tumor cells. As shown in Fig. S6A and S6B, western blot analysis of FAM172A showed the highest positive expression in Au NR@PAMAM-GX1/FAM172A + NIR treatment group.

### In vivo biocompatibility evaluation

**Fig. 8:**
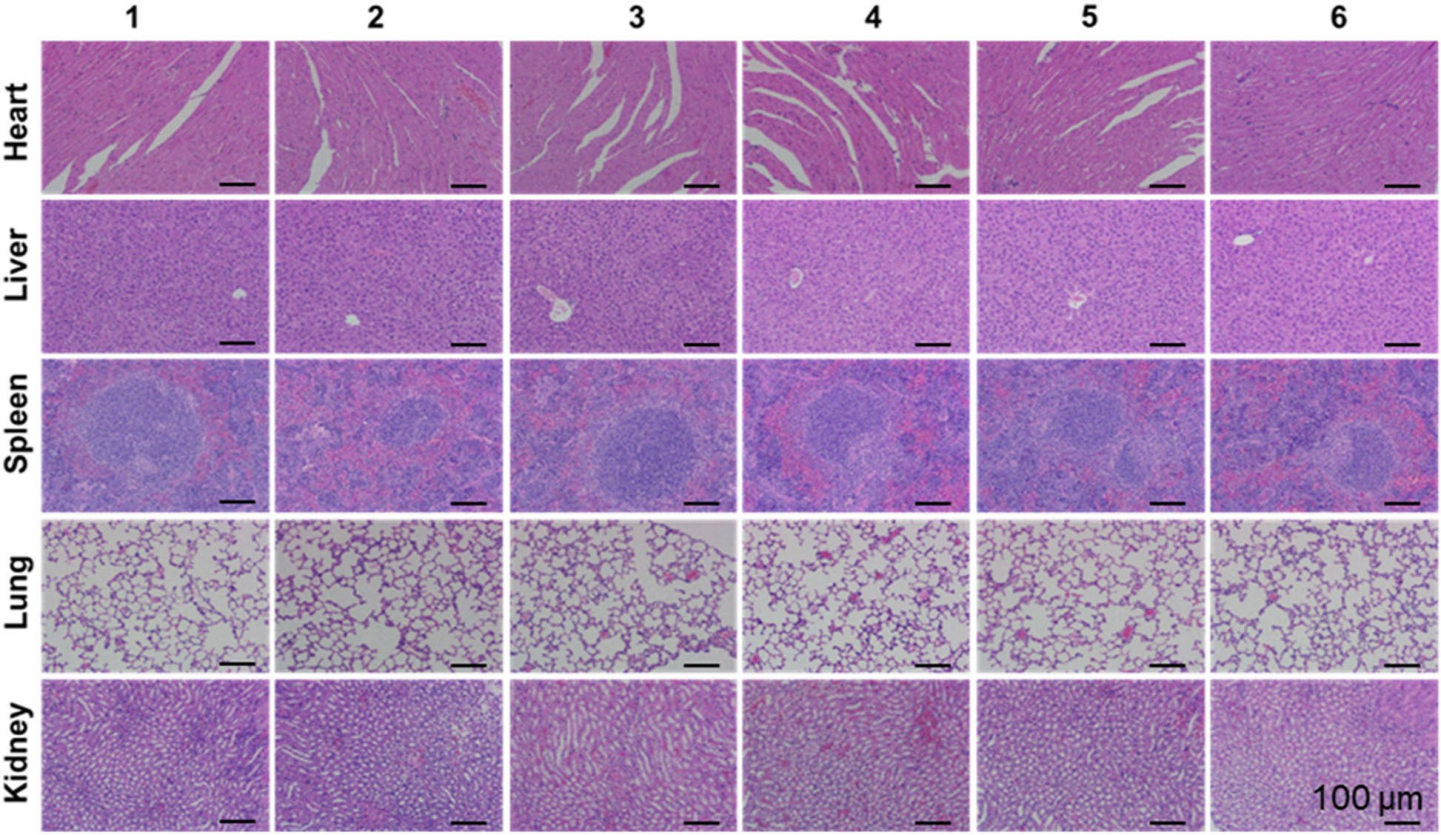
Histologic assessments of major organs in mice (200×) treated with different formulations. (1: PBS; 2: PBS + NIR; 3: Au NR@PAMAM-GX1; 4: Au NR@PAMAM-GX1/FAM172A; 5: Au NR@PAMAM-GX1 + NIR; 6: Au NR@PAMAM-GX1/FAM172A + NIR)

Biocompatibility is a prerequisite for the safe application of materials in nanomedicine. Therefore, the in vivo toxicity of the Au NR@PAMAM-GX1 complex to the main organs of mice were examined. As shown in Fig. 8, there is no obvious histological differences to the morphology and structure of the organs in each treatment group, which indicated that our nanocarriers have good biological safety.

## Conclusion

In summary, we have designed an innovative combination therapy platform with PTT therapy and gene therapy based on dendritic molecule-stabilized Au NSs. The partially thiolated third-generation (G3) poly (amidoamine) (PAMAM) dendrimers were combined with gold nanorods to form Au NR@PAMAM conjugate via Au-S bond. The GX1 polypeptide was combined with Au NR@PAMAM to obtain Au NR@PAMAM-GX1 through the amide reaction. Au NR@PAMAM-GX1 showed good cell compatibility within the studied concentration range and could be used as a carrier for delivering specific FAM172A to cancer cells. In addition, the Au NR@PAMAM-GX1 polyplexes can be serviced as a unique platform with enhanced PTT and gene therapy of cancer in vitro and in vivo. With the CT and thermal imaging functions of Au NR@PAMAM-GX1, which has huge potential in an integrated diagnosis and treatment platform for cancer treatment.

## Supplementary Information

**Additional file 1: Fig. S1.** TEM images of Au NR@CTAB. **Fig. S2.** Quantitative analysis of FITC intensity of Au NR@PAMAM or Au NR@PAMAM-GX at different time. **Fig. S3.** In vivo thermal images of tumor-bearing mice injected with Au NR@PAMAM, Au NR@PAMAM-GX1 and PBS, upon 808 nm-laser irradiations for different periods of time. **Fig. S4.** (A) In vivo CT images of tumor-bearing mice injected with Au NR@PAMAM and Au NR@PAMAM-GX1 from 0 min to 90 min. The white arrows indicate the tumor. (B) Quantification of Hounsfield unit (HU) value from CT images at 90 min. **Fig. S5.** Quantitative analysis of Ki67 expression at HCT-8 tumor tissues by immunohistochemical staining. **Fig. S6.** (A) Western bolt analysis of FAM172A. (B) FAM172A analyses for tissue expression. Data are expressed as the mean ± SD (*n* = 3).

## Data Availability

Not applicable.

## Abbreviations

PTT: Photothermal therapy
PAMAM: Poly(amidoamine) dendrimers
Au NRs: Gold nanorods
GT: Gene therapy
