# Supplementary material for: Dendrimer-modified gold nanorods as a platform for combinational gene therapy and photothermal therapy of tumors
Source: J Exp Clin Cancer Res. 2021 Sep 27;40:303. doi: 10.1186/s13046-021-02105-3 (PMC8477545; doi:10.1186/s13046-021-02105-3)
Supplement: Supplementary file 1 — Additional file 1: Fig. S1. TEM images of Au NR@CTAB. Fig. S2. Quantitative analysis of FITC intensity of Au NR@PAMAM or Au NR@PAMAM-GX at different time. Fig. S3. In vivo thermal images of tumor-bearing mice injected with Au NR@PAMAM, Au NR@PAMAM-GX1 and PBS, upon 808 nm-laser irradiations for different periods of time. Fig. S4. (A) In vivo CT images of tumor-bearing mice injected with Au NR@PAMAM and Au NR@PAMAM-GX1 from 0 min to 90 min. The white arrows indicate the tumor. (B) Quantification of Hounsfield unit (HU) value from CT images at 90 min. Fig. S5. Quantitative analysis of Ki67 expression at HCT-8 tumor tissues by immunohistochemical staining. Fig. S6. (A) Western bolt analysis of FAM172A. (B) FAM172A analyses for tissue expression. Data are expressed as the mean ± SD (n = 3). [file 13046_2021_2105_MOESM1_ESM.docx]

**Supporting information**

**Dendrimer-modified gold nanorods as a platform for combinational gene therapy and photothermal therapy of tumors**

Lili Ye ^1, †^, Yaoming Chen ^1, †^, Jizong Mao ^2^, Xiaotian Lei ^2^, Qian Yang ^2^, Chunhui Cui ^2, *^

^*^ Corresponding author: drcuich@163.com

^†^ Lili Ye and Yaoming Chen contributed equally to this work.

^1^ Department of Neuro-oncological Surgery, Zhujiang Hospital, Southern Medical University, Guangzhou, Guangdong Province, China

^2^ The Second School of Clinical Medicine, Southern Medical University, Guangzhou, Guangdong Province, China

**Figure Caption:**

**Fig. S1.** TEM images of Au NR@CTAB.

**Fig. S2.** Quantitative analysis of FITC intensity of Au NR@PAMAM or Au NR@PAMAM-GX at different time.

**Fig. S3.** *In vivo* thermal images of tumor-bearing mice injected with Au NR@PAMAM, Au NR@PAMAM-GX1 and PBS, upon 808 nm-laser irradiations for different periods of time.

**Fig. S4.** (A) *In vivo* CT images of tumor-bearing mice injected with Au NR@PAMAM and Au NR@PAMAM-GX1 from 0 min to 90 min. The white arrows indicate the tumor. (B) Quantification of Hounsfield unit (HU) value from CT images at 90 min.

**Fig. S5.** Quantitative analysis of Ki67 expression at HCT-8 tumor tissues by immunohistochemical staining.

**Fig. S6.** (A) Western bolt analysis of FAM172A. (B) FAM172A analyses for tissue expression. Data are expressed as the mean ± SD (n = 3).


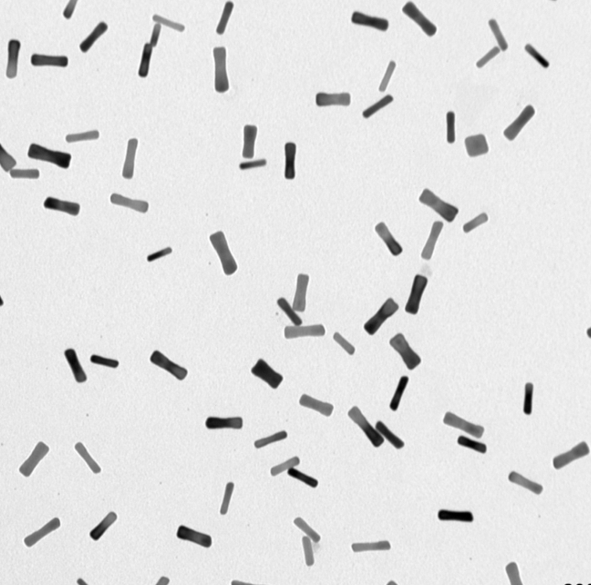


**100nm**

Fig. S1





Fig. S2


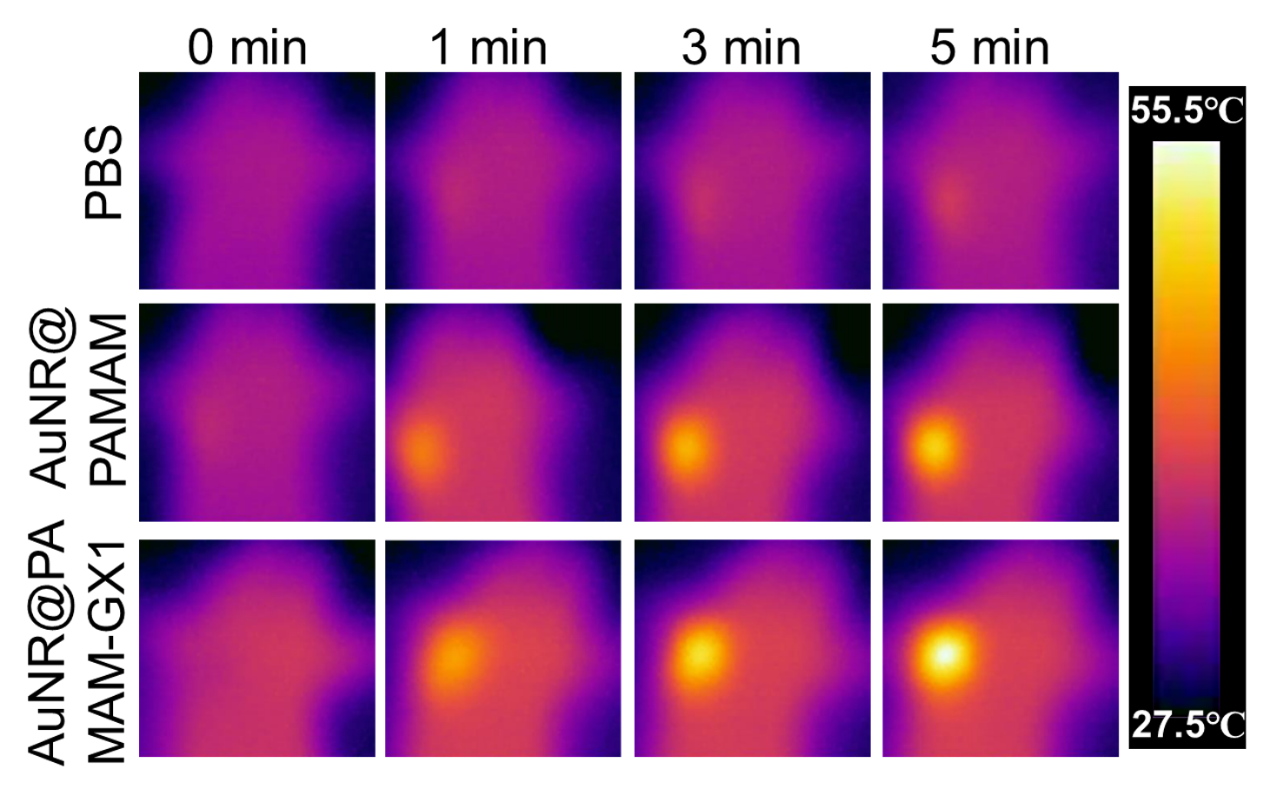


Fig. S3


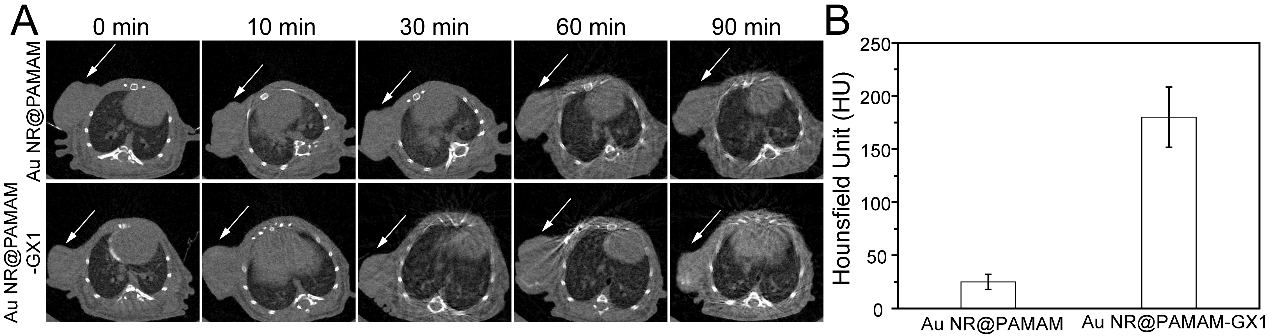


Fig. S4


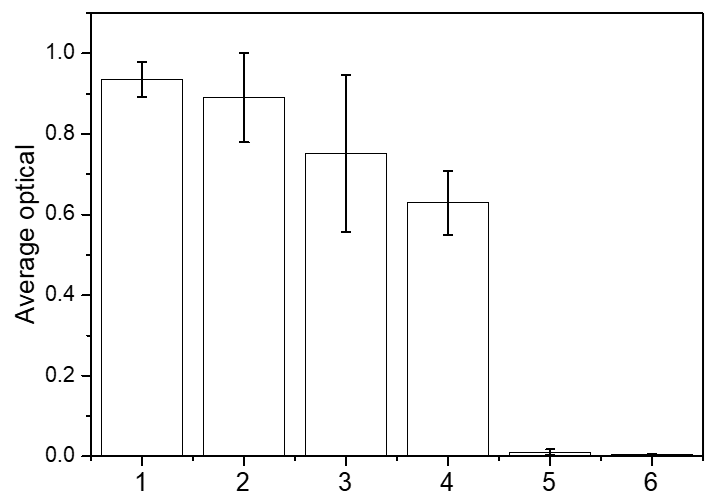


Fig. S5


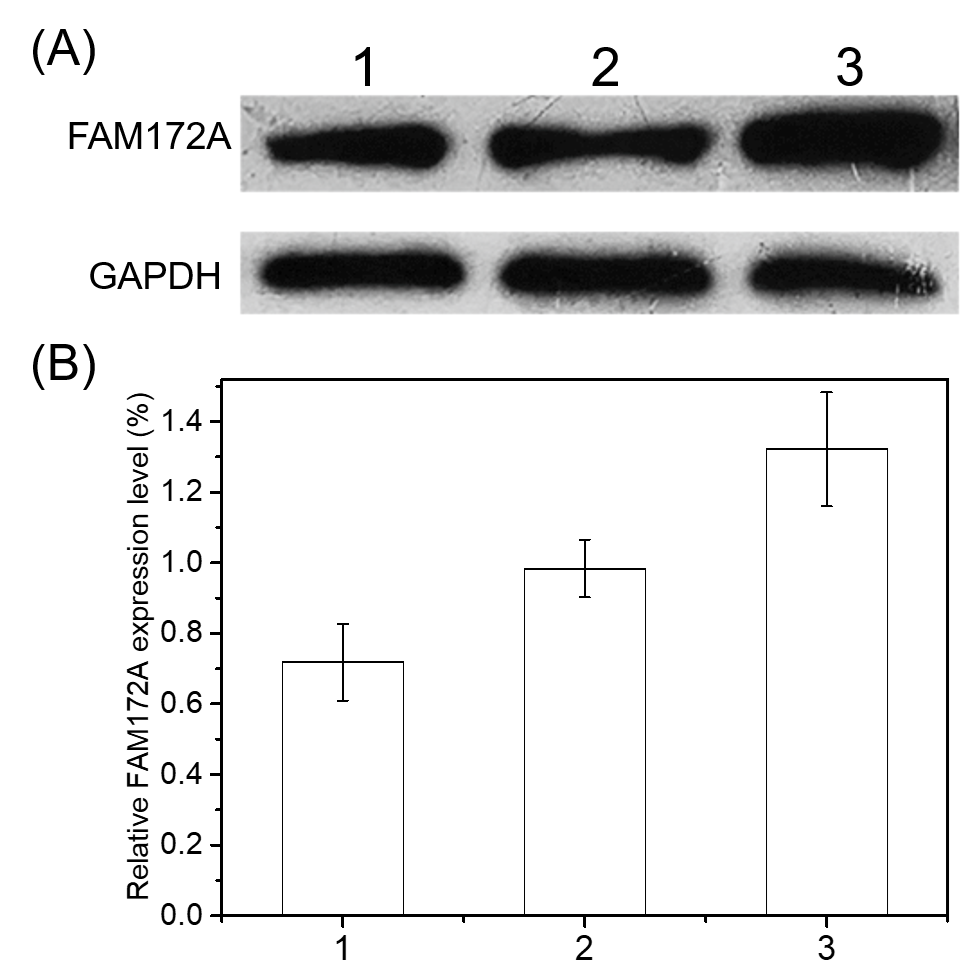


Fig. S6
